# Application of immunotherapy in advanced non-small cell lung cancer with hypertension: a multicenter retrospective analysis

**DOI:** 10.3389/fonc.2025.1621363

**Published:** 2025-09-12

**Authors:** Jingyi Yuan, Kaixin Lei, Quanling Kong, Tao Chang, Xinhang Gu, Juan Wang, Li-na He, Jiadi Gan, Bojiang Chen

**Affiliations:** ^1^ West China School of Medicine, West China Hospital, Sichuan University, Chengdu, China; ^2^ Department of Neurosurgery and Neurosurgery Research Laboratory, West China Hospital, Sichuan University, Chengdu, China; ^3^ Department of Radiation Oncology, Shandong Cancer Hospital and Institute, Shandong First Medical University and Shandong Academy of Medical Sciences, Jinan, Shandong, China; ^4^ Department of Radiation Oncology, The Affiliated Hospital of Qingdao University, Qingdao, Shandong, China; ^5^ Key Laboratory of Organ Regeneration and Transplantation of Ministry of Education, Institute of Immunology, The First Hospital, Jilin University, Changchun, China; ^6^ State Key Laboratory of Oncology in South People's Republic of China, Collaborative Innovation Center for Cancer Medicine, Sun Yat-sen University Cancer Center, Guangzhou, China; ^7^ Department of Respiratory and Critical Care Medicine, West China Hospital, Sichuan University, Chengdu, China; ^8^ Precision Medicine Research Center, Precision Medicine Key Laboratory of Sichuan Province, State Key Laboratory of Respiratory Health and Multimorbidity, West China Hospital, Sichuan University, Chengdu, Sichuan, China

**Keywords:** immune checkpoint inhibitor, hypertension, non-small cell lung cancer, PD-1/PD-L1 inhibitors, prognosis

## Abstract

**Importance:**

Immune checkpoint inhibitors (ICIs) have become the standard treatment for advanced non-small cell lung cancer (NSCLC). However, their prognostic role in NSCLC patients remains controversial. Hypertension (HTN) is an important risk factor for many cancers, but the pathogenesis underlying HTN in relation to cancer prognosis remains unclear.

**Objective:**

We aimed to investigate the possible association between HTN and prognosis in advanced NSCLC patients.

**Data sources:**

Data on advanced NSCLC patients receiving immunotherapy at stages IIIb, IIIc or IV were included.

**Study selection:**

Multicenter retrospective studies and trials reporting the use of immunotherapy were included. Main outcomes and measures: Progression-free survival (PFS) and overall survival (OS) were analyzed using Cox proportional hazards models and were estimated by the Kaplan-Meier method. Subgroup analysis on NSCLC hypertensive patients was pre-planned and was presented in the form of Forest Plot. Statistical software utilized for all analyses included statistical analysis system (SAS) V.9.4 and R version 4.2.2 (R Foundation for Statistical Computing).

**Results:**

Between January 2016 and June 2024, 1175 NSCLC patients receiving immunotherapy were enrolled, with 219 (18.6%) classified as hypertensive group and 956 (81.4%) classified as non-hypertensive group. Neutrophil count and ECOG = 2 showed a significant association with OS in univariate analysis (HR = 0.69, 95%Cl: 0.51 - 0.92, P = 0.012, and HR = 1.02, 95%Cl: 1.00 to 1.03, P = 0.008 respectively). In multivariate analysis, ECOG = 2 was significantly correlated with OS (HR = 0.73, 95%Cl: 0.54 to 0.98, P = 0.037) and PD - 1/PD-L1 had significant association with PFS (HR = 1.27, 95%Cl: 1.00 to 1.61, P = 0.050). OS was found significantly longer in non-hypertensive group than in hypertensive group (P = 0.049). No baseline indicator was found significant correlated with the survival prognosis of patients receiving immunotherapy in subgroup analysis.

**Conclusion and relevance:**

The non-hypertensive group was associated with a lower risk of mortality than hypertensive group. In subgroup analysis, no baseline indicator was observed a significant correlation with survival prognosis on OS and PFS in hypertensive patients. Our findings provided an important prognostic factor to improve the prognosis of advanced NSCLC patients receiving immunotherapy. Prospective randomized trials are needed to further validate these findings.

## 1 Background

Lung cancer is the malignant tumor with the highest morbidity and mortality in China, and indeed worldwide (1). Non-Small Cell Lung Cancer (NSCLC) is the most common pathological subtype, accounting for 85% of all lung cancers (2). However, 30%-40% of NSCLC patients are diagnosed at an advanced stage, having a poor prognosis (3). Immunotherapy plays an important role in the treatment of NSCLC patients. Recently, immune checkpoint inhibitors (ICIs) have brought a major breakthrough, especially those targeting the programmed death receptor (PD - 1) and programmed death ligand 1 (PD-L1), which have become the standard treatment for driver-negative advanced NSCLC. According to the American Society of Clinical Oncology (ASCO), continuing the exploration of immune strategies, optimization of existing immunotherapy protocols, and reduction of adverse outcomes of cancer treatment would be the focus of future research (4, 5).

Although immunotherapy has made a tremendous impact on patient survival in many high-incidence cancer indications (6), the response of patients to ICIs varies considerably. Patient-intrinsic factors (such as age, sex, body mass index (BMI) and human leukocyte antigen (HLA) genotype), tumor-intrinsic factors (such as the tumor-associated stroma) and environmental factors (such as the gut microbiota) may lead to the success or failure of the blockade of ICIs (7–10). In addition, controlling the gut microbiome may modulate the effectiveness of tumor immunotherapy (11). The key drivers of ICIs efficacy and patient survival have been summarized into five separate underlying factors: tumor mutation burden (TMB), T cell effective infiltration, transforming growth factor-beta (TGF-β) activity, prior treatment and proliferative potential (12). Therefore, different types of tumors can be systematically classified to better predict how patients would respond to ICIs.

The development of PD - 1 and PD-L1 has significantly extended the survival time of NSCLC patients, with higher response rates and lower incidence of side effects than anti-cytotoxic T lymphocyte-associated antigen-4 (CTLA - 4) (13, 14). Recent studies have shown that a variety of factors could affect the immunotherapy prognosis of advanced NSCLC patients. High BMI appeared to be associated with improved survival (15). CD161+CD127+CD8+T cells may be a key indicator of the poor prognosis in NSCLC patients with diabetes (16). ICIs, especially nivolumab combined with ipilimumab, might become one of the options for NSCLC patients with chronic kidney disease (CKD) (17). Moreover, the two most common adverse events of immunotherapy-related cardiotoxicity were myocarditis and pericarditis, which can lead to a high mortality rate (18). Beyond comorbidities, baseline inflammatory markers may critically guide ICI decisions. CRP level and neutrophil to lymphocyte ratio (NLR) may serve as a marker for the prognosis of advanced NSCLC patients receiving immunotherapy (19–21). Paradoxically, leukopenia (WBC<4×10^9^/L), which may have an association with malignancies, usually disqualified patients from clinical trials (22), though real-world data suggest comparable benefit (23–25).

Hypertension (HTN) remains a major chronic disease morbidity across the world. The prevalence of HTN continues to increase worldwide. The incidence of HTN in people aged 30–79 years doubled from 1990 to 2019, with 59% of women and 49% of men globally having previously diagnosed HTN in 2019 (26). Due to the common risk factors HTN and cancer shared, it might mask a causal relationship between high blood pressure (BP) and cancer (27). Some of the risk factors associated with HTN, such as obesity, type II diabetes mellitus, smoking, and a sedentary life-style, also increased the risk of cancer (28). Many studies have indicated that HTN was an important risk factor for certain cancers (29), such as breast cancer (30), lung cancer (31) and kidney cancer (32), which contributed to a larger mortality burden (33). The pathogenesis underlying HTN in relation to cancer prognosis remains unclear. Preclinical evidence suggested HTN may impair anti-PD-1 response through angiotensin II-mediated CD8+T cell suppression and vascular endothelial growth factor-driven myeloid-derived suppressor cell accumulation (34, 35). Moreover, previous murine models demonstrated calcium channel blockers (e.g., amlodipine) reverse tumor vasculature abnormalities and improve PD - 1 inhibitor efficacy (36). Yet human translational data remain conflicting: while renin-angiotensin-aldosterone system inhibitors correlate with prolonged OS in retrospective NSCLC cohorts (37), randomized trials show no benefit (38). This mechanistic uncertainty underscored the clinical importance to clarify HTN’s real-world impact in ICI-treated patients.

Nowadays, some studies have confirmed that HTN can affect the prognosis of patients receiving targeted therapy while few studies have focused on the correlation between HTN and immunotherapy of lung cancer. Therefore, in this study, we conducted a comparative analysis of 1175 patients diagnosed with advanced NSCLC and received immunotherapy at West China Hospital, West China Lung Cancer Center, Sichuan Cancer Hospital, Sichuan People’s Hospital, and Shandong Cancer Hospital from January 2016 to June 2024 to investigate the possible association between HTN and prognosis in advanced NSCLC patients.

## 2 Materials and methods

### 2.1 Patient materials

This multicenter prospective, observational study included all patients with pathologically confirmed advanced-stage NSCLC who were treated immunotherapy at West China Hospital of Sichuan University, West China Lung Cancer Center, Sichuan Cancer Hospital, Sichuan People’s Hospital, and Shandong Cancer Hospital from January 2016 to June 2024. Advanced-stage disease was defined as stage IIIb (N3M0), IIIc (N3M0) or IV (NanyM1) according to the AJCC Version 8 staging system. Pathologic confirmation was required, and the eligible histologies were as follows: squamous cell carcinoma, adenocarcinoma, large cell carcinoma, neuroendocrine carcinoma (NSCLC with neuroendocrine features or atypical carcinoids, but not small cell carcinoma) or NSCLC not otherwise specified. Data were collected into specific databases, with information on pathological type and features, previous medical and surgical history, diagnosis, metastasis and follow-up. The exclusion criteria included (1): incomplete treatment cycle (2); lack of available data on HTN (3); immunotherapy received after third-line treatment (4); no follow-up data (5); receipt of ICIs other than PD - 1/PD-L1 and (6) receipt of only neoadjuvant immunotherapy.

This study was approved by the Institutional Review Board of West China Hospital of Sichuan University (approval number 20241410), and all patients provided written informed consent.

### 2.2 Statistical methods

The main objectives were to assess the association between HTN and the prognosis of immunotherapy in advanced NSCLC patients with progression-free survival (PFS) and overall survival (OS) as the primary endpoints. Additionally, the direct efficacy of immunotherapy was evaluated, using complete response (CR), partial response (PR), stable disease (SD), progressive disease (PD), objective response rate (ORR) and disease control rate (DCR). PFS was defined as the time from the date of randomized treatment to the date of progression or death, whichever came first, while OS was defined as the time from the date of randomization to the date of death from any cause.

Baseline variables such as age, BMI, sex, underlying conditions, histology, smoking history, tumor stages, metastasis, medication, blood routine and PD-L1 TPS were included in patient characteristics. The chi-squared test (for categorical variables) or Wilcoxon rank sum test (for continuous variables) was used to observe the differences in baseline indicators between groups.

Patients with at least one evaluable endpoint (i.e. PFS or OS) were included in the present analysis. A possible different impact of HTN on prognosis was evaluated by means of survival curves. The Kaplan-Meier method was used to estimate OS and PFS, which was compared with log-rank tests.

Univariate and multivariate analyses cox proportional hazards models were used to determine the baseline indicators on OS and PFS without or with adjustment for other prognostic factors. All multivariate models included as covariates age, BMI, diabetes, coronary heart disease (CHD), lung metastasis, neutrophil count and medication. All the fitted models were stratified according to the enrolling center and results were presented as HR with their 95% Cl.

A further subgroup analysis was performed on NSCLC patients with HTN, including indicators such as age, BMI, diabetes, CHD, metastasis and type of immunotherapy drugs. The results of subgroup analysis were presented in the form of Forest Plot. Statistical software utilized for all analyses were statistical analysis system (SAS) V.9.4 and R version 4.2.2 (R Foundation for Statistical Computing). Statistical significance was defined as two-sided P <0.05. Our study fully aligned with methodological consensus (NCCN Biostatistics Guidelines 2023, https://www.nccn.org/guidelines/guidelines-detail?category=1&id=1450).

## 3 Result

### 3.1 Patients characteristics

**Figure 1:**
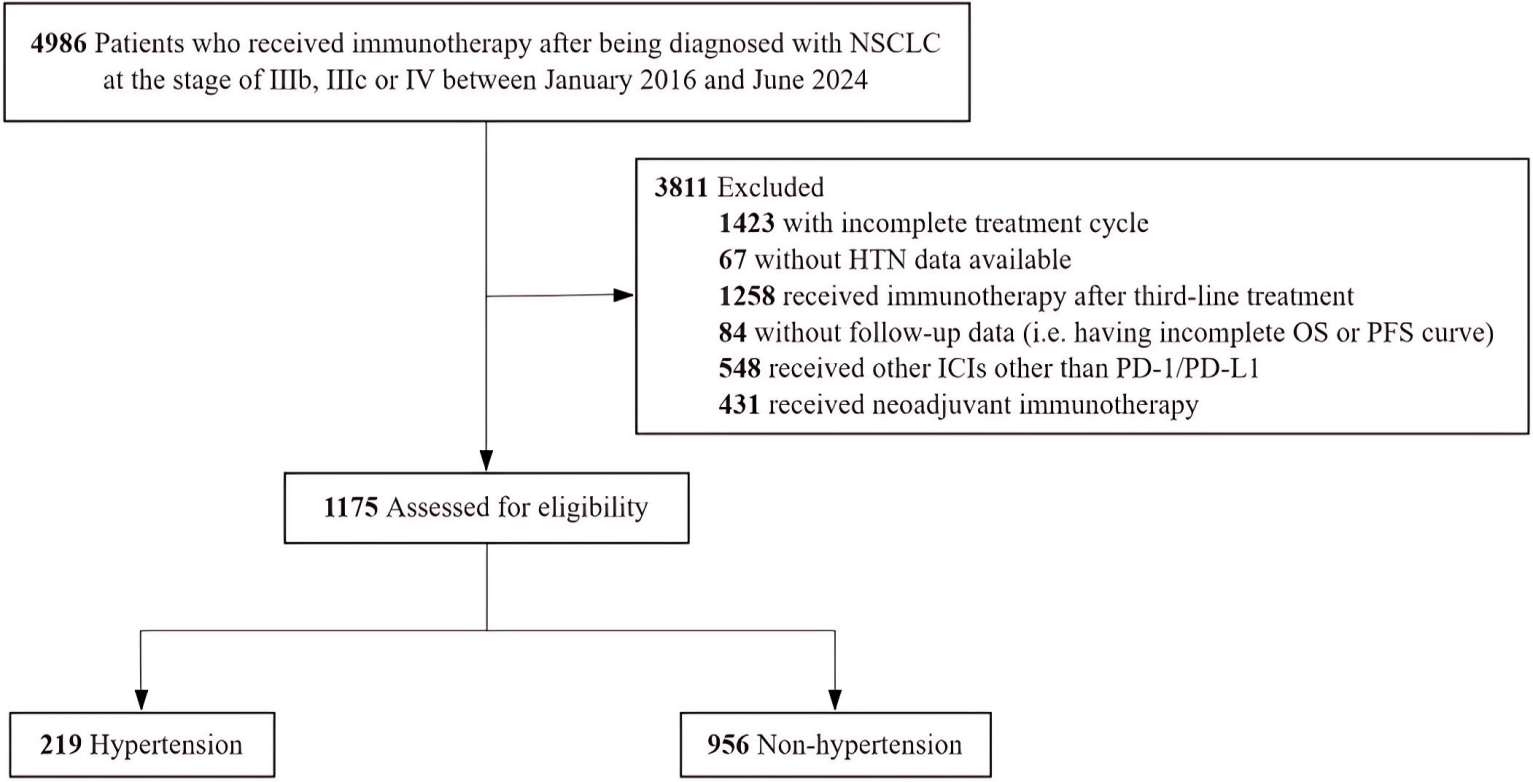
Flowchart of the Study Population. NSCLC indicates non-small cell lung cancer; HTN, hypertension; OS, overall survival; PFS, progression-free survival; ICIs, immune checkpoint inhibitors; PD-1, programmed death receptor and PD-L1, programmed death ligand1.

A total of 4896 patients were enrolled in the present study. 3811 patients (77.8%) were excluded based on the exclusion criteria (**Figure 1**). The remaining 1175 patients were included in the final analyses, classified into two groups: hypertensive group (n=219) and non-hypertensive group (n=956).

The demographic characteristics of the two groups were presented in **Table 1**. The hypertensive group had higher age and BMI than the non-hypertensive group (P<0.001). Among underlying conditions, the rates of diabetes and CHD in hypertensive group were higher than non-hypertensive groups (21.5% vs. 8.1%, P<0.001; 13.7% vs. 5.3%, P<0.001, respectively). Non-hypertensive group had a lower rate of metastasis than hypertensive group (14.4% vs. 6.8%, P = 0.011). Furthermore, a large proportion of the patients were male (78.7%), with the majority exhibiting ECOG scores of 1 - 2, and 645 patients (54.9%) were current or former smokers.

**Table 1:** Baseline characteristics of NSCLC patients categorized by hypertension and non-hypertension.

	Hypertension (n = 219)	Non-hypertension (n = 956)	*P* value
**Age median (range),y**	65.81 (64.88, 66.75)	61.06 (60.43, 61.7)	<0.001
**BMI mean (range)**	24.28 (23.85, 24.71)	23.12 (22.9, 23.34)	<0.001
**Sex n (%)**			0.613
Female	52 (23.7)	198 (20.7)	
Male	167 (76.3)	758 (79.3)	
**Smoking history (%)**			0.645
Never	105 (47.9)	425 (44.5)	
Ever	114 (52.1)	531 (55.5)	
**Histology (%)**			0.706
LUAD	130 (59.4)	598 (62.5)	
LUSC	72 (32.9)	309 (32.3)	
LCC	6 (2.7)	20 (2.1)	
Other	11 (5.0)	36 (3.8)	
**Underlying conditions (%)**			
Diabetes	47 (21.5)	77 (8.1)	<0.001
Kidney disease	24 (11.0)	102 (10.7)	0.992
Liver disease	22 (10)	124 (13.0)	0.496
Coronary heart disease	30 (13.7)	51 (5.3)	<0.001
**Tumor stages (%)**			0.301
IIIB, IIIC	37 (16.9)	123 (12.9)	
IVA, IVB	182 (83.1)	830 (86.8)	
**Metastasis (%)**			
None	15 (6.8)	138 (14.4)	0.011
Brain	40 (18.3)	181 (18.9)	0.974
Liver	188.2)	62 (6.5)	0.656
Bone	59 (26.9)	231 (24.2)	0.691
Lung/Peural	133 (60.7)	462 (48.3)	0.004
**EGFR mutation (n%)**	24 (11.0)	107 (11.2)	0.977
Plantlet count *10^9^/L mean (range)	259.48 (245.49, 273.48)	244.2 (238.25, 250.14)	0.08
Neutrophil count *10^9^/L mean (range)	6.48 (5.65, 7.31)	5.5 (5.13, 5.86)	<0.001
Lymphocyte count *10^9^/L mean (range)	1.87 (1.46, 2.27)	1.67 (1.5, 1.84)	0.01
Monocyte count *10^9^/L mean (range)	0.71 (0.59, 0.83)	0.69 (0.55, 0.83)	0.06
Leukocyte count *10^9^/L mean (range)	8.47 (7.85, 9.1)	7.45 (7.22, 7.69)	<0.001
PLR	192.01 (176.95, 207.08)	199.01 (191.05, 206.98)	0.627
NLR	4.55 (4.08, 5.03)	4.32 (4.06, 4.59)	0.292
LMR	3.07 (2.82, 3.32)	3.24 (2.97, 3.50)	0.820
**Medication n (%)**			0.035
IT	179 (81.7)	701 (73.3)	
IT+AA	40 (18.3)	255 (26.7)	
**PD-L1 TPS % (%)**			0.186
<1	39 (17.8)	194 (51.0)	
1~50	59 (26.9)	166 (17.4)	
≥50	32 (14.6)	108 (11.3)	

NSCLC, non-small cell lung cancer; BMI, body mass index; LUAD, lung adenocarcinomas; LUSC, lung squamous cell carcinomas; LCC, large cell carcinoma; EGFR, epidermal growth factor receptor; PLR, platelet to lymphocyte ratio; NLR, neutrophil to lymphocyte ratio; LMR, lymphocyte to monocyte ratio; IT, immunotherapy; AA, anti-angiogenesis inhibitors and TPS, tumor proportion score.

Bolded terms represented major categories; non-bolded terms beneath them indicated subcategories.

### 3.2 Impact of univariate on overall survival and progression-free survival

The results of the univariate analysis in OS and PFS were summarized in **Table 2**. Neutrophil count and ECOG = 2 showed a significant association with OS (HR = 0.69, 95%Cl: 0.51 - 0.92, P = 0.012, and HR = 1.02, 95%Cl: 1.00 - 1.03, P = 0.008 respectively). Neutrophil count was also the only one indicator that was associated with PFS (HR = 1.01, 95%Cl: 1.00 - 1.02, P = 0.050).

**Table 2:** Univariable Cox model for overall survival and progression-free survival in immunotherapy.

	Univariate			
	OS		PFS	
	HR (95%CI)	*P* value	HR (95%CI)	*P* value
**Age median (range), y**	0.99 (0.98 - 1.00)	0.396	0.99 (0.98 - 1.00)	0.183
**BMI mean (range)**	0.98 (0.94 - 1.01)	0.168	0.98 (0.94 - 1.01)	0.156
Male	1.11 (0.87 - 1.43)	0.396	1.09 (0.85 - 1.40)	0.505
**Smoking**	1.13 (0.92 - 1.38)	0.251	1.21 (0.99 - 1.49)	0.067
ECOG				
**0**	Reference		Reference	
**1**	1.10 (0.90 - 1.35)	0.278	0.96 (0.78 - 1.18)	0.697
**2**	0.69 (0.51 - 0.92)	0.012	0.80 (0.60 - 1.07)	0.134
Histology (%)				
LUSC	Reference		Reference	
LUAD	1.01 (0.82 - 1.24)	0.925	1.04 (0.85 - 1.27)	0.714
LCC	1.06 (0.59 - 1.90)	0.843	1.28 (0.72 - 2.28)	0.401
Underlying conditions (%)				
Diabetes	0.96 (0.71 - 1.31)	0.808	0.85 (0.62 - 1.16)	0.293
Kidney disease	0.89 (0.69 - 1.13)	0.338	0.87 (0.68 - 1.11)	0.268
Liver disease	0.98 (0.77 - 1.24)	0.844	0.88 (0.69 - 1.12)	0.292
Coronary heart disease	1.47 (0.84 - 2.56)	0.175	1.04 (0.60 - 1.82)	0.881
**Tumor stages (%)**		0.329		0.282
IIIB, IIIC	Reference		Reference	
IVA, IVB	0.90 (0.72 - 1.12)		0.89 (0.71 - 1.10)	
Metastasis (%)				
None	0.99 (0.76 - 1.28)	0.934	0.88 (0.68 - 1.14)	0.340
Brain	1.12 (0.84 - 1.50)	0.432	1.07 (0.80 - 1.44)	0.632
Liver	1.16 (0.83 - 1.62)	0.389	1.30 (0.93 - 1.82)	0.126
Bone	0.93 (0.73 - 1.18)	0.532	0.88 (0.69 - 1.11)	0.277
Lung/Peural	1.09 (0.89 - 1.34)	0.405	0.97 (0.79 - 1.19)	0.765
**EGFR mutation (n%)**	1.17 (0.82 - 1.67)	0.392	1.22 (0.85 - 1.74)	0.275
Plantlet count *10^9^/L mean (range)	1.00 (1.00 - 1.00)	0.851	1.00 (1.00 - 1.00)	0.651
Neutrophil count *10^9^/L mean (range)	1.02 (1.00 - 1.03)	0.008	1.01 (1.00 - 1.02)	0.050
Lymphocyte count *10^9^/L mean (range)	1.01 (0.98 - 1.03)	0.621	1.01 (0.98 - 1.04)	0.469
Monocyte count *10^9^/L mean (range)	1.03 (1.00 - 1.07)	0.058	1.03 (1.00 - 1.06)	0.057
Leukocyte count *10^9^/L mean (range)	1.02 (0.99 - 1.04)	0.160	1.01 (0.99 - 1.03)	0.384
PLR	1.00 (1.00 - 1.00)	0.647	1.00 (1.00 - 1.00)	0.924
NLR	1.01 (0.99 - 1.03)	0.551	1.00 (0.98 - 1.03)	0.747
LMR	1.00 (0.96 - 1.04)	0.889	0.99 (0.96 - 1.03)	0.770
**Medication n (%)**		0.477		0.682
IT	Reference		Reference	
IT+AA	1.09 (0.86 - 1.38)		1.05 (0.83 - 1.33)	
PD-L1 TPS % (%)				
<1	Reference		Reference	
1~50	0.82 (0.62 - 1.07)	0.140	0.85 (0.65 - 1.11)	0.233
≥50	1.21 (0.89 - 1.65)	0.217	1.33 (0.98 - 1.81)	0.069

BMI, body mass index; OS, overall survival; PFS, progression-free survival; ECOG, eastern cooperative oncology group; LUAD, lung adenocarcinomas; LUSC, lung squamous cell carcinomas; LCC, large cell carcinoma; EGFR, epidermal growth factor receptor; PLR, platelet to lymphocyte ratio; NLR, neutrophil to lymphocyte ratio; LMR, lymphocyte to monocyte ratio; IT, immunotherapy; AA, anti-angiogenesis inhibitors and TPS, tumor proportion score.

Bolded terms represented major categories; non-bolded terms beneath them indicated subcategories.

However, the PD-L1 TPS between 1% and 50% and more than 50% were associated with increased HR of 0.82 (95%Cl: 0.62 - 1.07, P = 0.140) and 1.21 (95%Cl: 0.89 - 1.65, P = 0.217), respectively, suggesting decreased OS compared to the PD-L1 TPS less than 1%.

### 3.3 Impact of multivariate on overall survival and progression-free survival

The results of the multivariable Cox model on OS and PFS were illustrated in **Table 3**, including indicators such as age, BMI, ECOG = 2, underlying condition (diabetes and CHD), lung/pleural metastasis, neutrophil count, medication and PD - 1/PD-L1.

**Table 3:** Multivariable Cox model for overall survival and progression-free survival in immunotherapy.

	Multivariate			
	OS		PFS	
	HR (95%CI)	*P* value	HR (95%CI)	*P* value
**Age median (range), y**	0.99 (0.98 - 1.00)	0.199	0.99 (0.98 - 1.00)	0.148
**BMI mean (range)**	0.98 (0.95 - 1.02)	0.294	0.98 (0.94 - 1.01)	0.190
**ECOG = 2**	0.73 (0.54 - 0.98)	0.037	–	–
Diabetes	0.97 (0.71 - 1.34)	0.156	0.88 (0.64 - 1.20)	0.412
Coronary heart disease	1.51 (0.85 - 2.68)	0.156	0.97 (0.55 - 1.72)	0.913
Lung/Peural metastasis	1.05 (0.89 - 1.30)	0.623	0.96 (0.78 - 1.18)	0.683
Neutrophil count *10^9^/L mean (range)	1.01 (1.00 - 1.03)	0.087	1.01 (1.00 - 1.02)	0.067
**Medication n (%)**		0.791		0.893
IT	Reference		Reference	
IT+AA	1.04 (0.81 - 1.32)		1.01 (0.80 - 1.30)	
**PD-1/PD-L1**		–		0.050
PD-L1	–		Reference	
PD-1	–		1.27 (1.00 - 1.61)	

BMI, body mass index; OS, overall survival; PFS, progression-free survival; ECOG, eastern cooperative oncology group; IT, immunotherapy; AA, anti-angiogenesis inhibitors; PD - 1, programmed death receptor and PD-L1, programmed death ligand 1.

Bolded terms represented major categories; non-bolded terms beneath them indicated subcategories.

A multivariate logistic regression analysis demonstrated that ECOG = 2 was significantly correlated with OS (HR = 0.73, 95%Cl: 0.54 to 0.98, P = 0.037) while PD - 1/PD-L1 had significant association with PFS (HR = 1.27, 95%Cl: 1.00 to 1.61, P = 0.050). No impact of other variables was detected on OS and PFS.

### 3.4 Immunotherapy survival analysis on overall survival and progression-free survival between hypertensive group and non-hypertensive group

**Figure 2:**
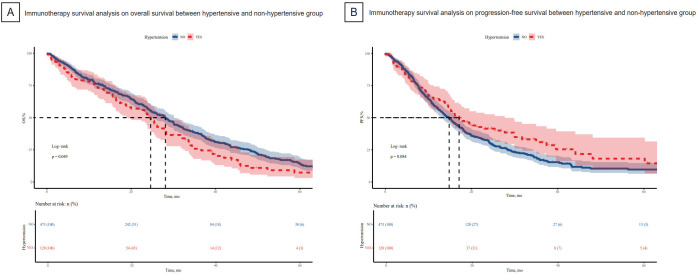
Kaplan_meier curves for survival prognosis between hypertensive group and non-hypertensive group were shown. **(A)** OS was significantly longer in non-hypertensive group than the hypertensive group. **(B)** PFS in hypertensive group was not better group. OS, overall survival.

We employed the Kaplan-Meier method to examine survival differences between hypertensive and non-hypertensive groups. OS was significantly longer in non-hypertensive group than in hypertensive group (P = 0.049, **Figure 2A**). Conversely, survival prognosis of immunotherapy of hypertensive group was not better than non-hypertensive group (P = 0.084, **Figure 2B**).

**Figure 3:**
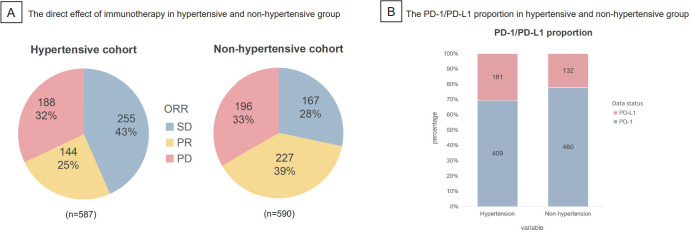
The direct effect and PD-1/PD-L1 proportion in hypertensive and non-hypertensive group were shown. **(A)** ORR in hypertensive group was 25%, much lower than the 39% in non-hypertensive group. **(B)** Staked bar plot presented the distribution of PD-1/PD-L1 in hypertensive and non-hypertensive group, indication that PD-1 made up the majority both in hypertensive (69.3%) and non hypertensive group (77.7%). ORR, objective response rate (SD, stable disease; PR, partial response; PD, progressive disease.

The direct effects of immunotherapy in hypertensive and non-hypertensive groups were illustrated respectively in the pie charts (**Figure 3A**). ORR in hypertensive group (25%) was far lower than that in non-hypertensive group (39%), which indicated a potential of better treatment in non-hypertensive group. Furthermore, stacked bar plot presented the PD - 1/PD-L1 proportion in hypertensive and non-hypertensive groups (**Figure 3B**). PD - 1 dominated both in hypertensive (69.3%) and non-hypertensive group (77.7%), which may suggest that PD - 1 had a stronger effect on immunotherapy prognosis than PD-L1.

### 3.5 Subgroup analysis on overall survival and progression-free survival

**Figure 4:**
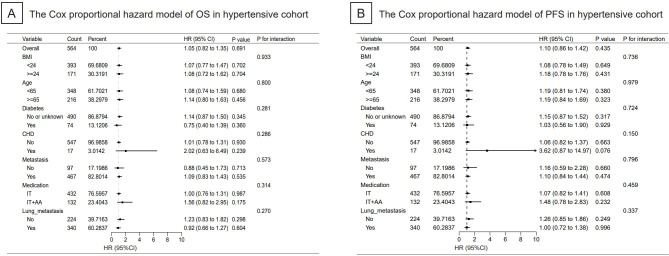
Cox proportional hazard models of significant independent predictive factors associated with progression-free survival and overall survival were shown. As illustrated in the form of Forest Plot, there was no significance in OS and PFS analysis between the indicators and the survival prognosis of immunotherapy. OS, overall survival; PFS, progression-free-survival; BMI, body mass index; CHD, coronary heart disease; IT, immunotherapy and AA, anti-angiogenesis inhibitors.

A cohort of 564 NSCLC hypertensive patients were included in the subgroup analysis on OS and PFS. However, there was no significance in OS (**Figure 4A**) and PFS (**Figure 4B**) analysis between the baseline indicators and the survival prognosis of patients receiving immunotherapy.

## 4 Discussion

With the emergence of ICIs, these drugs have made significant breakthroughs in the treatment of NSCLC and have rapidly become a standard treatment for driver-negative patients. Currently, PD - 1/PD-L1 is recognized as an important biomarker used in clinical practice (39, 40). However, complex clinical conditions, such as baseline characteristics and underlying diseases, may influence the effectiveness of immunotherapy and affect the identification of NSCLC patients who benefit from it. Indeed, identifying indicators that influence the prognosis of immunotherapy in NSCLC patients may be a promising strategy to improve survival and reduce mortality.

In our univariate and multivariate analyses, PD - 1/PD-L1 was significantly associated with PFS, while ECOG = 2 was associated with OS, consistent with previous studies that ECOG PS score≥2 has important prognostic value in NSCLC patients receiving ICIs treatment (41). In a previous single-arm trial assessing the safety and efficacy of PD - 1 inhibitors in advanced NSCLC, PD - 1 blockade (Camrelizumab)-activated neoantigen-specific cellular therapy (aNASCT) demonstrated safety and immunogenicity in NSCLC patients, suggesting its promising potential in cancer immunotherapy (42). Another observational study indicated that PD - 1 inhibitors were widely administered to NSCLC patients with stage III and IV extensively metastatic lung cancer, showing significantly longer PFS and OS (43–46). Anti-PD-1 antibody alone or combined with chemotherapy has become the standard of care for advanced NSCLC patients (47). Consistent with previous studies, our study showed a significant correlation between PD - 1/PD-L1 and PFS. However, we did not observe a significant association between PD - 1/PD-L1 and OS. This discrepancy may be due to variations in the study population, the type of PD - 1/PD-L1 utilized, the sample size, and the method of obtaining the results.

Our study found that, among advanced NSCLC patients who received immunotherapy, the non-hypertensive group was associated with a lower risk of mortality than hypertensive group. Additionally, no baseline indicator was observed a significant correlation with survival prognosis in hypertensive patients. HTN was reported that it may lead to a poor survival for advanced NSCLC patients with type 2 diabetes mellitus (48). Nevertheless, in our study, we didn’t see this correlation, which was not consistent with previous research. The results of studies on the correlation between HTN and the prognosis of targeted therapy in NSCLC patients varies. Some indicated some targeted drug (such as bevacizumab) was associated with an increased risk of HTN development in NSCLC patients (49), which led to the poor prognosis of treatment. Others showed that HTN in bevacizumab-treated patients with metastatic NSCLC led to significantly improved responses (50). The occurrence of HTN may be an important clinical indicator predicting the efficacy of third-line targeted drug treatment in NSCLC patients (51). However, no specific large-scale studies of the correlation between HTN and the prognosis of immunotherapy in NSCLC patients was observed.

Due to the fact that there was no previous studies on the relevant pathogenesis, we hypothesized the reasons why there was a significant correlation between HTN and the prognosis of immunotherapy in NSCLC patients were as follows. HTN was reported to be independently associated with increased risk of both VEGF-related adverse vascular events (AVEs) and discontinuation due to VEGF-related AVEs, which may be an important factor to affect the prognosis of immunotherapy (52).

Voltage-gated calcium channels (VGCCs) increased BP in HTN by promotin vasoconstriction, while abnormally regulated calcium channels in cancer may drive tumor cell’s proliferation, migration, and immune escape (36). In other words, some anti-hypertensive drugs may affect the prognosis of NSCLC immunotherapy, which needed to be confirmed by further studies.

Overall, our data suggested that further research would be needed to elucidate the relationship between HTN and the prognosis of immunotherapy in NSCLC patients.

## 5 Limitations

The findings of this study had to be seen in light of the following limitations. Firstly, this was a retrospective study, and the inherent drawbacks of retrospective studies made some selection bias inevitable, while the lack of original imaging data in a proportion of patients made it difficult to further explore the prognostic impact of NSCLC patients receiving immunotherapy. Secondly, the data used were not specifically collected for the purposes of this study, but were derived from existing clinical practice information, not allowing for a randomized clinical trial (RCT). Thirdly, the results of this study were based on data from the population in five hospitals. The moderate sample size of NSCLC patients receiving immunotherapy limited the ability to draw a definitive conclusion and the generalizability to other populations. However, our study provided the preliminary evidence for future large-scale and prospective trials. Fourthly, data of PD-L1 expression were sparse and some of the PD-L1 status could not be determined from this database. There was a possibility that patients with low or negative PD-L1 expression were more likely to receive conventional chemotherapy, which were excluded from this study. Finally, while CRP, ESR and cytokines were not measured due to retrospective constraints, we analyzed guideline-recommended hematologic indices (e.g., NLR, PLR and LMR) and adjusting for direct inflammation proxies (e.g., neutrophil count, ECOG). Future studies should integrate multiplex cytokine profiling. At the same time, this study did not further investigate the effects of different types of anti-hypertensive drugs on the prognosis of NSCLC patients receiving immunotherapy. Further studies should be based on samples using the same ICI drugs (PD - 1/PD-L1 inhibitors) and classified according to the types of anti-hypertensive drugs to investigate its impact on immunotherapy prognosis.

## 6 Conclusion

Our study is the first large-scale retrospective multicenter study to investigate the association between HTN and the prognosis of immunotherapy in advanced NSCLC patients. In our study, the non-hypertensive group was associated with a lower risk of mortality compared to the hypertensive group. In subgroup analysis, no baseline indicator was observed a significant correlation with survival prognosis on OS and PFS in hypertensive patients. Our findings provide an important prognostic factor to improve the prognosis of advanced NSCLC patients receiving immunotherapy. Prospective randomized trials are warranted to further validate these findings.

## Data Availability

The original contributions presented in the study are included in the article/supplementary material, further inquiries can be directed to the corresponding author Jiadi Gan (Med_ganjd@163.com).

## References

[1] Han B Zheng R Zeng H Wang S Sun K Chen R. Cancer incidence and mortality in China, 2022. Zhonghua Zhong Liu Za Zhi. (2024) 46:221–31. doi: 10.3760/cma.j.cn112152-20240119-00035, PMID: 38468501

[2] Herbst R Morgensztern D Boshoff C. The biology and management of non-small cell lung cancer. Nature. (2018) 553:446–54. doi: 10.1038/nature25183, PMID: 29364287

[3] Travis WD Brambilla E Nicholson AG Yatabe Y Austin JHM Beasley MB. The 2015 world health organization classification of lung tumors: impact of genetic, clinical and radiologic advances since the 2004 classification. J Thorac Oncol. (2015) 10:1243–60. doi: 10.1097/JTO.0000000000000630, PMID: 26291008

[4] Remon J Passiglia F Ahn MJ Barlesi F Forde PM Garon EB. Immune checkpoint inhibitors in thoracic Malignancies: review of the existing evidence by an IASLC expert panel and recommendations. J Thorac Oncol. (2020) 15:914–47. doi: 10.1016/j.jtho.2020.03.006, PMID: 32179179

[5] Hendriks LE Kerr KM Menis J Mok TS Nestle U Passaro A. Electronic address: clinicalguidelines@esmo.org. Oncogene-addicted metastatic non-small-cell lung cancer: ESMO Clinical Practice Guideline for diagnosis, treatment and follow-up. Ann Oncol. (2023) 34:339–57. doi: 10.1016/j.annonc.2022.12.009, PMID: 36872130

[6] Korman AJ Garrett-Thomson SC Lonberg N. The foundations of immune checkpoint blockade and the ipilimumab approval decennial. Nat Rev Drug Discov. (2022) 21:509–28. doi: 10.1038/s41573-021-00345-8, PMID: 34937915

[7] Kalbasi A Ribas A. Tumour-intrinsic resistance to immune checkpoint blockade. Nat Rev Immunol. (2020) 20:25–39. doi: 10.1038/s41577-019-0218-4, PMID: 31570880 PMC8499690

[8] Chowell D Morris LGT Grigg CM Weber JK Samstein RM Makarov V. Patient HLA class I genotype influences cancer response to checkpoint blockade immunotherapy. Science. (2018) 359:582–7. doi: 10.1126/science.aao4572, PMID: 29217585 PMC6057471

[9] Pitt JM Vétizou M Daillère R Roberti MP Yamazaki T Routy B. Resistance mechanisms to immune-checkpoint blockade in cancer: tumor-intrinsic and -extrinsic factors. Immunity. (2016) 44:1255–69. doi: 10.1016/j.immuni.2016.06.001, PMID: 27332730

[10] Fessler J Matson V Gajewski TF. Exploring the emerging role of the microbiome in cancer immunotherapy. J Immunother Cancer. (2019) 7:108. doi: 10.1186/s40425-019-0574-4, PMID: 30995949 PMC6471869

[11] Sivan A Corrales L Hubert N Williams JB Aquino-Michaels K Earley ZM. Commensal Bifidobacterium promotes antitumor immunity and facilitates anti–PD-L1 efficacy. Science. (2015) 350:1084–9. doi: 10.1126/science.aac4255, PMID: 26541606 PMC4873287

[12] Usset J Rosendahl Huber A Andrianova MA Batlle E Carles J Cuppen E. Five latent factors underlie response to immunotherapy. Nat Genet. (2024) 56:2112–20. doi: 10.1038/s41588-024-01899-0, PMID: 39266764 PMC11525176

[13] Zhang D Huang J Zhang C Guan Y Guo Q. Progress on PD - 1/PD-L1 checkpoint inhibitors in lung cancer. Zhongguo Fei Ai Za Zhi. (2019) 22:369–79. doi: 10.3779/j.issn.1009-3419.2019.06.07, PMID: 31196371 PMC6580087

[14] Havel JJ Chowell D Chan TA. The evolving landscape of biomarkers for checkpoint inhibitor immunotherapy. Nat Rev Cancer. (2019) 19:133–50. doi: 10.1038/s41568-019-0116-x, PMID: 30755690 PMC6705396

[15] Kichenadasse G Miners JO Mangoni AA Rowland A Hopkins AM Sorich MJ. Association between body mass index and overall survival with immune checkpoint inhibitor therapy for advanced non-small cell lung cancer. JAMA Oncol. (2020) 6:512–8. doi: 10.1001/jamaoncol.2019.5241, PMID: 31876896 PMC6990855

[16] Qu J Li Y Wu B Shen Q Chen L Sun W. CD161+CD127+CD8+ T cell subsets can predict the efficacy of anti-PD-1 immunotherapy in non-small cell lung cancer with diabetes mellitus. Oncoimmunology. (2024) 13:2371575. doi: 10.1080/2162402X.2024.2371575, PMID: 38952673 PMC11216103

[17] Matsumoto H Maezawa Y Ohara G Shiozawa T Masuko H Satoh H. Long-term control by immune checkpoint inhibitors in a lung cancer patient with chronic kidney disease. Klin Onkol. (2024) 38:375–9. doi: 10.48095/ccko2024375, PMID: 39516036

[18] Sławiński G Wrona A Dąbrowska-Kugacka A Raczak G Lewicka E. Immune checkpoint inhibitors and cardiac toxicity in patients treated for non-small lung cancer: A review. Int J Mol Sci. (2020) 21:7195. doi: 10.3390/ijms21197195, PMID: 33003425 PMC7582741

[19] Li Y Zhang Z Hu Y Yan X Song Q Wang G. Pretreatment neutrophil-to-lymphocyte ratio (NLR) may predict the outcomes of advanced non-small-cell lung cancer (NSCLC) patients treated with immune checkpoint inhibitors (ICIs). Front Oncol. (2020) 10:654. doi: 10.3389/fonc.2020.00654, PMID: 32656072 PMC7324627

[20] Tong W Xu H Tang J Zhao N Zhou D Chen C. Inflammatory factors are associated with prognosis of non-small cell lung cancer patients receiving immunotherapy: a meta-analysis. Sci Rep. (2024) 14:26102. doi: 10.1038/s41598-024-76052-2, PMID: 39478006 PMC11525588

[21] Fujimoto A Toyokawa G Koutake Y Kimura S Kawamata Y Fukuishi K. Association between pretreatment neutrophil-to-lymphocyte ratio and immune-related adverse events due to immune checkpoint inhibitors in patients with non-small cell lung cancer. Thorac Cancer. (2021) 12:2198–204. doi: 10.1111/1759-7714.14063IF:2.3Q2, PMID: 34173724 PMC8327687

[22] Weber JS Hodi FS Wolchok JD Topalian SL SChadendorf D Larkin J. Safety profile of nivolumab monotherapy: A pooled analysis of patients with advanced melanoma. J Clin Oncol. (2017) 35:785–92. doi: 10.1200/JCO.2015.66.1389, PMID: 28068177

[23] Hara H Mizusawa J Hironaka S Kato K Daiko H Abe T. Influence of preoperative chemotherapy-induced leukopenia on survival in patients with esophageal squamous cell carcinoma: exploratory analysis of JCOG9907. Esophagus. (2021) 18:41–8. doi: 10.1007/s10388-020-00752-7, PMID: 32514753

[24] Dechow T Riera-Knorrenschild J Hackanson B Janssen J Schulz H Oppermann U. First-line nab-paclitaxel plus carboplatin for patients with advanced non-small cell lung cancer: Final results of the NEPTUN study. Int J Cancer. (2023) 153:141–52. doi: 10.1002/ijc.34467, PMID: 36757197

[25] Kha VV Ly TH Duy PDT Hoa PT Cong BT. The prognostic significance of pretreatment white blood cell and platelet counts for survival outcome in primary lung cancer. Mater Sociomed. (2024) 36:97–102. doi: 10.5455/msm.2024.36.97-102, PMID: 38590595 PMC10999138

[26] NCD Risk Factor Collaboration (NCD-RisC). Worldwide trends in hypertension prevalence and progress in treatment and control from 1990 to 2019: a pooled analysis of 1201 population-representative studies with 104 million participants. Lancet. (2021) 398:957–80. doi: 10.1016/S0140-6736(21)01330-1, PMID: 34450083 PMC8446938

[27] Kidoguchi S Sugano N Tokudome G Yokoo T Yano Y Hatake K. New concept of onco-hypertension and future perspectives. Hypertension. (2021) 77:16–27. doi: 10.1161/HYPERTENSIONAHA.120.16044, PMID: 33222548

[28] Cohen JB Geara AS Hogan JJ Townsend RR. Hypertension in cancer patients and survivors: epidemiology, diagnosis, and management. JACC CardioOncol. (2019) 1:238–51. doi: 10.1016/j.jaccao.2019.11.009, PMID: 32206762 PMC7089580

[29] Sahni G. Onco-hypertension: changing paradigm of treating hypertension in patients with cancer. J Clin Oncol. (2023) 41:958–63. doi: 10.1200/JCO.22.01875, PMID: 36332165

[30] Han H Guo W Shi W Yu Y Zhang Y Ye X. Hypertension and breast cancer risk: a systematic review and meta-analysis. Sci Rep. (2017) 7:44877. doi: 10.1038/srep44877, PMID: 28317900 PMC5357949

[31] Lindgren A Pukkala E Nissinen A Tuomilehto J. Blood pressure, smoking, and the incidence of lung cancer in hypertensive men in North Karelia, Finland. Am J Epidemiol. (2003) 158:442–7. doi: 10.1093/aje/kwg179, PMID: 12936899

[32] Everatt R Kuzmickienė I Brasiūnienė B Vincerževskienė I Intaitė B Cicėnas S. Postdiagnostic use of antihypertensive medications and survival in colorectal, lung, corpus uteri, melanoma and kidney cancer patients with hypertension. BMC Cancer. (2025) 25:38. doi: 10.1186/s12885-024-13273-8, PMID: 39780067 PMC11707882

[33] Arima H Barzi F Chalmers J. Mortality patterns in hypertension. J Hypertens. (2011) 29 Suppl 1:S3–7. doi: 10.1097/01.hjh.0000410246.59221.b1, PMID: 22157565

[34] Zhang R Yin H Yang M Liu J Zhen D Zhang Z. Advanced progress of the relationship between renin-angiotensin-aldosterone system inhibitors and cancers. J Hypertens. (2024) 42:1862–73. doi: 10.1097/HJH.0000000000003836, PMID: 39248142

[35] Deluce J Maj D Verma S Breadner D Boldt G Raphael J. Efficacy and toxicity of combined inhibition of EGFR and VEGF in patients with advanced non-small cell lung cancer harboring activating EGFR mutations: A systematic review and meta-analysis. Am J Clin Oncol. (2023) 46:87–93. doi: 10.1097/COC.0000000000000976, PMID: 36661266

[36] Sheng Y Qiao C Zhang Z Shi X Yang L Xi R. Calcium channel blocker lacidipine promotes antitumor immunity by reprogramming tryptophan metabolism. Adv Sci (Weinh). (2025) 12:e2409310. doi: 10.1002/advs.202409310, PMID: 39585774 PMC11744582

[37] Wei J Zhou Z Xu Z Zeng S Chen X Wang X. Retrospective clinical study of renin-angiotensin system blockers in lung cancer patients with hypertension. PeerJ. (2019) 7:e8188. doi: 10.7717/peerj.8188, PMID: 31844581 PMC6910116

[38] Rachow T Schiffl H Lang SM. Risk of lung cancer and renin-angiotensin blockade: a concise review. J Cancer Res Clin Oncol. (2021) 147:195–204. doi: 10.1007/s00432-020-03445-xIF:2.8Q2, PMID: 33231730 PMC7684567

[39] Carbone DP Reck M Paz-Ares L. CheckMate 026 investigators. First-Line Nivolumab Stage IV Recurrent Non-Small-Cell Lung Cancer. N Engl J Med. (2017) 376:2415–26. doi: 10.1056/NEJMoa1613493, PMID: 28636851 PMC6487310

[40] Reck M. Pembrolizumab as first-line therapy for metastatic non-small-cell lung cancer. Immunotherapy. (2018) 10:93–105. doi: 10.2217/imt-2017-0121, PMID: 29145737

[41] Dall’Olio FG Maggio I Massucci M. ECOG performance status ≥2 as a prognostic factor in patients with advanced non small cell lung cancer treated with immune checkpoint inhibitors-A systematic review and meta-analysis of real world data. Lung Cancer. (2020) 145:95–104. doi: 10.1016/j.lungcan.2020.04.027, PMID: 32417680

[42] Qiao Y Hui K Hu C Wang M Sun W Liu L. Efficacy and safety of PD - 1 blockade-activated neoantigen specific cellular therapy for advanced relapsed non-small cell lung cancer. Cancer Immunol Immunother. (2025) 74:60. doi: 10.1007/s00262-024-03906-z, PMID: 39751937 PMC11699067

[43] Wang C Liang N Qiao L Wu Y Zhang J Zhang Y. Clinical features and prognosis analysis of stage III/IV patients with lung cancer after treatment with toripalimab: A real-world retrospective. J Cancer Res Ther. (2024) 20:2021–8. doi: 10.4103/jcrt.jcrt_500_24, PMID: 39792412

[44] Luan T Shen PX Zhang YH Xie XH Lin XQ Liu M. Annual therapeutic advances in advanced non-small cell lung cancer in 2024. Zhonghua Jie He He Hu Xi Za Zhi. (2025) 48:72–7. doi: 10.3760/cma.j.cn112147-20241103-00655, PMID: 39757100

[45] Yoshida K Watanabe K Nishimura T Ikushima H Ohara S Takeshima H. Improvement in survival in patients with advanced non-small cell lung cancer. Anticancer Res. (2025) 45:295–305. doi: 10.21873/anticanres.17417, PMID: 39740836

[46] Poddubskaya E Suntsova M Lyadova M Luppov D Guryanova A Lyadov V. Biomarkers of success of anti-PD-(L)1 immunotherapy for non-small cell lung cancer derived from RNA- and whole-exome sequencing: results of a prospective observational study on a cohort of 85 patients. Front Immunol. (2024) 15:1493877. doi: 10.3389/fimmu.2024.1493877, PMID: 39723204 PMC11669362

[47] Gelibter A Tuosto L Asquino A Siringo M Sabatini A Zizzari IG. Anti-PD1 therapies induce an early expansion of Ki67+CD8+ T cells in metastatic non-oncogene addicted NSCLC patients. Front Immunol. (2024) 15:1483182. doi: 10.3389/fimmu.2024.1483182, PMID: 39744631 PMC11688303

[48] Zeng X Zeng D Cheng J Xu C Sun C Long H. Influence of hypertension on the survival of non-small cell lung cancer patients with type 2 diabetes mellitus. Med Sci Monit. (2020) 26:e921676. doi: 10.12659/MSM.921676, PMID: 32305990 PMC7191951

[49] Chen J Lu Y Zheng Y. Incidence and risk of hypertension with bevacizumab in non-small-cell lung cancer patients: a meta-analysis of randomized controlled trials. Drug Des Devel Ther. (2015) 9:4751–60. doi: 10.2147/DDDT.S87258, PMID: 26316712 PMC4547635

[50] Yan LZ Dressler EV Adams VR. Association of hypertension and treatment outcomes in advanced stage non-small cell lung cancer patients treated with bevacizumab or non-bevacizumab containing regimens. J Oncol Pharm Pract. (2018) 24:209–17. doi: 10.1177/1078155217690921, PMID: 29284349

[51] Shi J Chen G Wang H Wang X Han B Li K. Occurrence of hypertension during third-line anlotinib is associated with progression-free survival in patients with squamous cell lung cancer (SCC): A *post hoc* analysis of the ALTER0303 trial. Thorac Cancer. (2021) 12:2345–51. doi: 10.1111/1759-7714.14076, PMID: 34273139 PMC8410552

[52] Naito T Minegishi Y Shiraishi H Hoshino T Maeda J Yokota T. Influence of background cardiovascular risk factors on VEGF inhibitor-related adverse vascular events in patients with non-small cell lung cancer: a retrospective study. J Cancer Res Clin Oncol. (2023) 149:12435–42. doi: 10.1007/s00432-023-05092-4, PMID: 37439826 PMC11796653

